# Genetic polymorphisms of *PRL*, *DGAT1*, *FSHR*, and *GH* genes and their associations with milk and reproduction traits in Egyptian Buffalo (*Bubalus bubalis*)

**DOI:** 10.1186/s12864-025-12019-5

**Published:** 2025-10-13

**Authors:** Abdelfatah R. Zaghloul, Maher H. Khalil, Mahmoud M. Iraqi, Amin M.S. Amin, Ayman G. EL Nagar

**Affiliations:** 1 Department of Animal Production, Faculty of Agriculture, Benha University, Moshtohor, Tukh, Kalyoubia Governorate 13736 Egypt; 2 Animal Production Research Institute, Agricultural Research Center, Ministry of Agriculture, Dokki, Egypt

**Keywords:** Egyptian buffalo, Lactation traits, Reproduction traits, Molecular association, *PRL*, *DGAT1*, *FSHR* and *GH* genes, PCR-RFLP

## Abstract

**Background & objectives:**

Genetic polymorphisms in key candidate genes such as *PRL*, *DGAT1*, *FSHR*, and *GH* play a pivotal role in regulating lactation and reproductive traits, making them critical markers for breeding programs in buffalo. Molecular characterization and associations of *PRL*, *DGAT1*, *FSHR* and *GH* candidate genes with test day milk yield (TDMY), fat yield (TDFY), protein yield (TDPY), somatic cell score (TDSCS), age at first calving (AFC), days open (DO) and calving interval (CI) in Egyptian buffalo.

**Methods:**

Lactation and reproduction records were obtained from Egyptian buffaloes reared in three experimental herds affiliated with the Animal Production Research Institute (APRI), Agricultural Research Center (ARC), Ministry of Agriculture, Egypt. For molecular characterization and association analysis of candidate genes with the studied traits, blood samples were collected from a total of 286 animals (both males and females). Specifically, 101 animals were successfully genotyped for *PRL* and *DGAT1* genes, 98 females and 71 males for *FSHR* gene, and 103 females and 71 males for *GH* gene. PCR-RFLP technique using *XbaI* restriction enzyme for *PRL* gene and using *AluI* restriction enzyme for *DGAT1*,* FSHR* and *GH* genes was used for animal’s genotyping.

**Results:**

The generalised least square means (GLSMs) for AA genotype of *PRL* gene were superior relative to GG genotype, being 6.0 vs. 5.3 kg for TDMY, 390 vs. 340 g for TDFY, 290 vs. 220 g for TDPY and 2.47 vs. 2.50 for TDSCS, while the GLSM for GG genotype was favourable than AA genotype for all the studied reproductive traits. The CC genotype of *FSHR* gene was superior relative to GG and GC genotypes for all lactation traits, being 6.8 vs. 5.6 and 5.7 kg for TDMY, 380 vs. 360 and 350 g for TDFY, 290 vs. 220 and 230 g for TDPY and 2.41 vs. 2.48 and 2.45 for TDSCS, while the CC genotype was favourable than GG and GC genotypes for AFC, DO and CI. For *GH* gene, TC genotype was superior compared to CC genotype, being 6.1 vs. 5.6 kg for TDMY, 390 vs. 350 g for TDFY, 290 vs. 220 g for TDPY, 2.41 vs. 2.45 for TDSCS, 34.4 vs. 37.6 *mo* for AFC, 95 vs. 107 *d* for DO and 377 vs. 399 *d* for CI.

**Conclusion:**

The significant molecular associations detected between AA genotype of *PRL* gene, CC genotype of *FSHR* gene and TC genotype of *GH* gene and lactation and reproduction traits may be helpful for marker-assisted selection programs aiming to improve lactation traits and reproduction performance in Egyptian buffalo.

**Supplementary Information:**

The online version contains supplementary material available at 10.1186/s12864-025-12019-5.

## Introduction

Domestic water buffalo (*Bubalus bubalis*) are classified into two classes as river buffalo (*Bubalus bubalis bubalis*) and swamp buffalo (*Bubalus bubalis carabanesis*) [1]. The molecular studies for populations of buffaloes in terms of DNA sequencing, *SNP*s and PCR-RFLP techniques, computer software and bioinformatics methodology have been facilitated to identify the molecular markers and candidate genes controlling lactation and reproduction traits [2–9]. These molecular markers could be used in marker assisted selection programs to improve the selection response of lactation and reproduction traits in buffalo. The improvement of reproduction performance in buffaloes by traditional selection programs is difficult task, due to long generation interval and low heritability estimates for reproduction traits [3]. In Egyptian buffalo, the reproduction efficiency is greatly influenced by infertility disorders such as anoestrus, inactive ovaries, repeat service [10].

In the last decade, the molecular characterizations for some functional candidate genes were found in different buffalo studies. Among the various genes, prolactin gene (*PRL*) was mapped on chromosome 2 [11, 12], and this gene was molecularly characterized in Nili-Ravi buffalo [4, 13], Murrah buffalo [14], Anatolian water buffalo [15, 16] and Egyptian buffalo [17, 18]. On the other hand, Diacylglycerol O-Acyltransferase 1 gene (*DGAT1)* was mapped on chromosome 15 and this gene was molecularly characterized in Anatolian buffalo [19], in Murrah buffalo [3, 20, 21], Mediterranean buffalo [20] and Iraqi buffalo [22]. Also, Follicle-stimulating hormone receptor gene (*FSHR*) was mapped on chromosome number 12 and it was molecularly investigated in Egyptian buffalo [23, 24]. Moreover, the buffalo *FSHR* gene is comprised of an open reading frame of 2085 bp which encodes a 695 amino acid protein [25]. Moreover, growth hormone gene (*GH*) is located on chromosome number 3 and the structure of this gene in buffalo species was unknown [15, 26–29]. Some studies were performed to characterize molecularly this gene in Egyptian buffalo [23], Anatolian water buffalo [15], and Simeulue buffalo [29].

On worldwide, the molecular buffalo studies have shown that *PRL*,* DGAT1*,* FSHR* and *GH* genes could be used as candidate genes in the genetic improvement programs for lactation and reproduction traits of buffalo in Pakistan [4], in China [30], in Turkey [15, 16], and in India [14]. Also, the Egyptian buffalo molecular studies verified that *PRL*, *DGAT1*, *FSHR* and *GH* genes are considered as important candidate genes that are molecularly associated with milk yields and compositions, reproduction and fertility, semen, body weights and gains in Egyptian buffalo [8, 10, 23, 24, 31]. However, *PRL* gene is known to have various biological functions such as water and electrolyte balance, growth, development, immunity and reproduction function [32]. Also, *PRL* gene plays a vital role in mammalian reproduction, glandular development, milk secretion, and expression of milk protein. In Murrah buffalo, Singh et al. [33] found that *PRL* gene is an important candidate gene known to be associated with milk production traits as well as somatic cell counts (SCC). The Egyptian studies have shown that *FSHR* gene is considered as an important candidate gene for lactation, reproduction, fertility and semen traits in Egyptian buffalo [23, 24, 31, 34]. Shafik et al. [34] found significant association between *FSHR* gene and calving interval, days open, days in milk, total milk yield and 305-day milk yield. Regarding *GH* gene, this gene can be used as a candidate gene for the genetic improvement of growth traits in buffalo since it is known to have various biological functions such as water and electrolyte balance, milk production and reproduction functions [23, 35]. Despite of the economic relevance of Egyptian buffalo, a species of significant national importance for milk and meat production in Egypt, the studies assessing the genetic characterization of candidate genes and their associations with productive and reproductive traits are scarce. The objectives of the current study were: (1) to identify SNPs in four candidate genes (*PRL*,* DGAT1*, *FSHR* and *GH*) in Egyptian buffalo breed, (2) to apply PCR-RFLP technique using *XbaI* and *AluI* restriction enzymes in genotyping the SNP located in the promoter regions of these genes, and (3) to associate the SNP detected in *PRL*,* DGAT1*,* FSHR* and *GH* candidate genes with milk production and reproduction traits.

## Materials and methods

### Ethical statement and animal rights

All experimental procedures involving animal handling and treatment were approved by the Research Ethics Committee of the Faculty of Agriculture, Benha University, Egypt (Approval number: REC-FOABU 0.3/00041), with a confirmation that all procedures were done by the relevant guidelines and regulations. The animals were kept under the standard operating procedures of Benha University. The study was reported in accordance with the checklist of recommendations developed by Animal Research: Reporting of in Vivo Experiments (ARRIVE). This work was performed in the project No. 33,531, entitled “A genomic Approach to Improve Production and Reproduction Traits in Egyptian Buffalo” funded by the Science, Technology & Innovation Funding Authority (STDF), Ministry of Higher Education and Scientific Research, Egypt.

### Buffalo herds, management and studied traits

Three experimental buffalo herds nominated as El-Nattafe El-Gadid (NG), El-Nattafe El-Kadim (NK) and El-Gimmeza (EG), belonging to the Animal Production Research Institute (APRI), Agriculture Research Center (ARC), Ministry of Agriculture, Egypt were used in this study. The herds of NG and NK are in Kafr El-Sheikh Governorate, while EG herd is in Gharbia Governorate. The management system followed in all the three experimental stations was the same according to APRI recommendations. Buffaloes were kept under semi-open sheds; heifers were joined for the first service when reaching 24 months of age or 330 kg body weight. Buffaloes were naturally mated in a group-mating system. Rectal palpation was applied to check pregnancy at 60 days post-mating. Milking was practiced twice a day at 7 AM and 4 PM throughout the lactation period. Buffaloes were fed Egyptian Berseem (*Trifolium alexandrinum*) along with varying amounts of integrated concentrate feed mixture (48% decorticated cotton seed cake, 21% wheat bran, 20% maize, 5% rice polish, 3% molasses, 2% Limestone, and 1% sodium chloride) according to APRI feeding routine. The calves were weighed immediately after birth, fed colostrum for the first three days after birth at 3% of their body weight and then weighed monthly. Buffaloes were dried off two months before the expected day of calving. The abnormal lactations or reproduction records affected by diseases or having missing birth dates, dry off dates or yields were excluded. More details regarding the management procedures and feeding regimes followed in these herds, more details are described intensively by Zaghloul et al. [36]. A primary objective of these experimental stations is the conservation of Egyptian buffalo, a species of significant national importance for both milk and meat production. To achieve this, APRI operates a calf-raising facility where selective breeding has been conducted continuously for four decades. The resulting offspring are then distributed to the research stations to form mating groups. Throughout this process, APRI carefully considers relationships, avoiding matings between close relatives and animals with common grandparents to minimize the increase of inbreeding. Therefore, the calculated mean inbreeding rate in NG, NK and EG herds were 0.003, 0.005 and 0.003, respectively. In the studied experimental herds, means of total milk yield were 1674.34, 1595.17 and 1486.50 kg for NG, NK and EG, respectively. Moreover, the averages of lactation period length were 201.12, 200.58 and 229.17 days for NG, NK and EG, respectively.

The studied lactation and reproductive traits were test-day milk yield (TDMY), fat yield (TDFY), protein yield (TDPY), somatic cell score (TDSCS), age at first calving (AFC), days open (DO) and calving interval (CI). Records of test-day milk (TD) were collected following an alternative AM: PM monthly recording scheme. Analyses of milk composition were conducted using automated infrared absorption spectrophotometry (Milk-o-Scan™; Foss Electric, Hillerφd, Denmark) at the Dairy Services Unit, International Livestock Management Training Center (ILMTC), APRI, Egypt. Moreover, test-day somatic cell score (TDSCS) was derived from test-day somatic cell count (TDSCC) data using log-transformation method achieve an approximate normal distribution [37]. The formula used for TDSCS calculation was TDSCS = log_2_ (TDSCC/100) + 3. Age at first calving (AFC), days open (DO) and calving interval (CI) records were collected from database file of APRI, Agriculture Research Center, Ministry of Agriculture, Egypt. All the known genetic relationships among the animals were considered in the data analysis.

### Blood sampling, DNA extraction, PCR amplification and genotyping by PCR-RFLP

All the blood samples were collected from live animals non-invasively under the normal farm conditions, *i.e.* no animals were slaughtered for all purposes of the study. For DNA extraction, blood samples were collected from the jugular veins of 200 buffalo cows and 86 buffalo bulls. Following sterilization of the puncture site, 10 to 15 ml of blood per animal was drawn from fully conscious animals without anaesthesia, ensuring minimal distress. Blood samples were collected into EDTA-coated vacutainer tubes, immediately labelled, placed in ice-cooled boxes, and transported to the laboratory for further processing. The primers used in the amplification process for *PRL* [18], *DGAT1* [19], *FSHR* [38] and *GH* [15] genes were presented in Table 1. The protocols of DNA extraction, PCR amplification and PCR-RFLP technique using *XbaI* restriction enzyme for *PRL* gene and using *AluI* restriction enzyme for *DGAT1*,* FSHR* and *GH* genes were previously illustrated by Zaghloul et al. [39]. Concerning *DGAT1* gene, a 411 *bp* fragment was amplified using a primer forward 5´-GCACCATCCTCTTCCTCAAG-3´and reverse 5´-GGAAGCGCTTTCGG ATG-3´. PCR amplification conditions for *DGAT1* gene were as follows: the thermal cycling conditions were composed of a pre-denaturation step at 95 °C for 15 min, followed by 35 cycles of denaturation at 94 °C for one minute, annealing at 60˚C for one minute, elongation at 72 °C for one minute and then final extension at 72˚C for ten minutes.

**Table 1:** Primer sequence and annealing temperatures for *PRL*, *DGAT1*,* FSHR* and *GH* genes

Gene and analysed exon	CN^a^	Primer sequences	PCR Product size (bp)	Annealing temp (ºC per time, s)	Reference
*PRL (exon 4)*	2	F: AGGTTAGGAGGATAG R: TTAGTCAAGTTAGATACCG	678	50.5/60	[18]
*DGAT1 (exon 8)*	15	F: GCACCATCCTCTTCCTCAAG R: GGAAGCGCTTTCGGATG	411	60/60	[19]
*FSHR (part of exon 10)*	12	F: CTGCCTCCCTCAAGGTGCCCCTC R: AGTTCTTGGCTAAATGTCTTAGGGGG	306	60/30	[38]
*GH (exon 5)*	3	F: GCTGCTCCTGAGGGCCCTTC R: CATGACCCTCAGGTACGTCTCCG	211	62/60	[15]

^a^*CN *Chromosome number

### Molecular parameters to characterize *PRL*, *DGAT1*, *FSHR* and *GH* genes

From the 286 collected blood samples, a total of 101 buffalo cows (30 from NG and 71 from NK) were successfully genotyped for *PRL* gene. For *DGAT1* gene, the same numbers of animals were used. For *FSHR* gene, a total of 169 animals were used: 98 buffalo cows (33 from NG, 44 from NK and 21 from EG) for both characterization and association studies and 71 buffalo bulls for characterization only. For *GH* gene, a total of 174 animals (51 from NG, 97 from NK and 26 from EG) were used: 103 females and 71 males. These differences between the number of blood samples and the number of animals successfully genotyped for each gene may be attributed to DNA quality and/or amplification success. The genetic diversity of *PRL*,* DGAT1*, *FSHR* and *GH* genes were assessed in each herd separately and across all herds by calculating the effective number of alleles (*Ne*), Chi-square values for Hardy-Weinberg equilibrium (*HWE*) and the observed (*Ho*) and expected (*He*) heterozygosities using GENALEX software version 6.5 [40]. The following equations were used to estimate the previous parameters:

$$\:\varvec{N}\varvec{e}=\frac{1}{{\sum\:}_{\varvec{i}=1}^{\varvec{n}}{\varvec{p}}_{\varvec{i}}^{2}}\:\:\:\:\:\:\:\:\:\:\:\:\:\:\:\varvec{H}\varvec{o}=\frac{\varvec{N}\varvec{o}.\:\varvec{o}\varvec{f}\:\varvec{h}\varvec{e}\varvec{t}\varvec{e}\varvec{r}\varvec{o}\varvec{z}\varvec{y}\varvec{g}\varvec{o}\varvec{s}\varvec{i}\varvec{t}\varvec{y}\:}{\varvec{n}}\:\:\:\:\:\:\:\:\:\:\:\:\:\:\:\varvec{H}\varvec{e}=1-{\sum\:}_{\varvec{i}=1}^{\varvec{n}}{\varvec{p}}_{\varvec{i}}^{2}$$

The polymorphism information content (PIC) was calculated using CERVUS software version 3 [41] as:

$$\:\varvec{P}\varvec{I}\varvec{C}=1-{\sum\:}_{\varvec{i}=1}^{\varvec{n}}{\varvec{p}}_{\varvec{i}}^{2}-{\sum\:}_{\varvec{i}=1}^{\varvec{n}-1}.{\sum\:}_{\varvec{j}=\varvec{i}+1}^{\varvec{n}}2{\varvec{p}}_{\varvec{i}}^{2}{\varvec{p}}_{\varvec{j}}^{2}\:$$

Where P_i_ = the frequency of the i^th^ allele, P_j_ = the frequency of the j^th^ allele and n = the number of alleles.

### Models for detecting polymorphic associations

For association analysis, the number of lactation records and genotyped animals gathered in NG and NK herds as well as the number of reproduction records obtained in NG, NK and EG herds for the studied buffaloes are presented in Table 2. The CFC software version 1.0 was used to examine the pedigree file for relationship issues [42]. The renumf90 software was used to renumber the data [43]. Using the TM software [44], a Bayesian inference of Gibbs Sampling Algorithm was used to estimate the variance components for the random effects of the studied traits which consequently used to solve the corresponding mixed model equations by using the PEST software to obtain the generalized least-squares means (GLSM) for different SNP genotypes [45]. The molecular associations between *PRL*, *FSHR* and *GH* genotypes and milk production and reproduction traits were assessed in each herd separately and across herds. To detect the molecular associations between *PRL*,* FSHR* and *GH* genes with lactation traits (TDMY, TDFY, TDPY and TDSCS), the following repeatability single-trait animal model was used:

**Table 2:** Number of records and genotyped animals (between brackets) used in molecular association analyses for lactation and reproduction traits in NG, NK and EG herds

Gene and traits	NG herd	NK herd	EG herd	All herds
*PRL* gene:				
Lactation traits	324 (30)	705 (71)	-	1029 (101)
Reproduction traits	143 (33)	210 (43)	100 (25)	453 (101)
*FSHR* gene:				
Lactation traits	435 (33)	767 (65)	-	1202 (98)
Reproduction traits	151 (33)	196 (44)	84 (21)	431 (98)
*GH* gene:				
Lactation traits	324 (34)	705 (69)	-	1029 (103)
Reproduction traits	143 (33)	224 (45)	93 (25)	460 (103)

$$\mathrm y\;=\;\mathbf{Xb}\boldsymbol\;+\;{\mathbf Z}_{\mathbf a}{\mathbf u}_{\mathbf a}\boldsymbol\;+\;{\mathbf Z}_{\mathbf p}{\mathbf u}_{\mathbf p}\boldsymbol\;+\;\mathrm e\;\left(\mathrm{Model}\;1\right)$$

where: **y** = the observation on the lactation trait; **b** = vector of the fixed effects including herd-year-test-day (271 levels), parity (5 levels; 1^st^ (20 animals), 2^nd^ (35 animals), 3^rd^ (29 animals), 4^th^ (26 animals) and 5^th^ to 7^th^ (40 animals), season of calving (4 levels), SNP genotypes effects (AA and GG genotypes for *PRL* gene; GG, GC and CC genotypes for *FSHR* gene; TC and CC genotypes for *GH* gene) and covariable of days in milk (DIM); **u**_**a**_ = the vector of random additive genetic effects of the buffalo cow; u_p_ = the vector of random permanent non-additive environmental effects of the buffalo cow; **X**,** Z**_**a**_ and **Z**_**p**_ = design matrices for fixed effects, random additive genetic effects and random permanent environmental effects, respectively; **e** = vector of random error.

To detect the molecular associations between *PRL*,* FSHR* and *GH* genes with reproduction traits (DO and CI), the following single-trait animal model was used:

$$\mathrm y\;=\;\mathbf{Xb}\boldsymbol\;+\;{\mathbf Z}_{\mathbf a}{\mathbf u}_{\mathbf a}\boldsymbol\;+\;{\mathbf Z}_{\mathbf p}{\mathbf u}_{\mathbf p}\boldsymbol\;+\;\mathrm e\;\left(\mathrm{Model}\;2\right)$$

where: **y** = the observation on DO or CI; **b** = the vector of fixed effects of herd-year-season of calving (380 levels), parity (4 levels; 1^st^ (103 animals), 2^nd^ (75 animals), 3^rd^ (46 animals) and 4^th^ to 7^th^ (28 animals), SNP genotypes effects, **u**_**a**_, **u**_**p**_, **X**,** Z**_**a**_ and **Z**_**p**_ are the same as defined previously in Model 1. Data of AFC was analysed using the same Model 2 after excluding the fixed effect of parity and the random non-additive permanent environmental effects. Data of each single herd was analysed separately using the same animal model after deleting the herd effect from Models 1&2.

## Results

### Polymorphic characterization of *PRL* gene

**Fig. 1:**
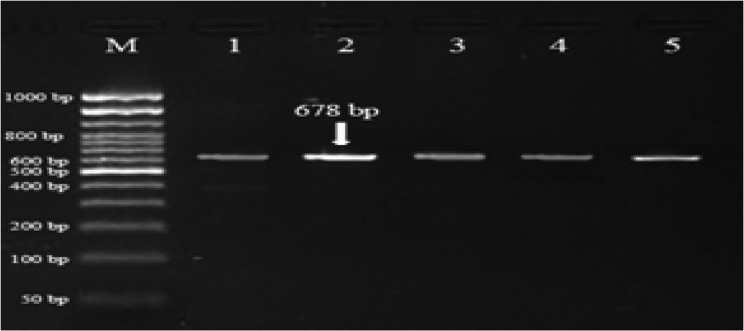
Gel electrophoresis showing the PCR product of *PRL* gene in Egyptian buffalo, 678 *bp* band. M is 50 *bp* ladder DNA molecular marker

**Fig. 2:**
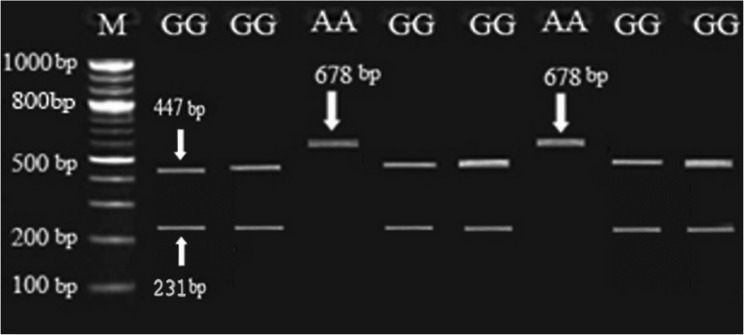
Gel electrophoresis showing the PCR-RFLP of the SNP identified in *PRL* gene in Egyptian buffalo. The genotypes are indicated at the top of each lane, 678 *bp*, 447 *bp* and 231 *bp* bands, M is 100 *bp* ladder DNA molecular marker

Digestion of 678 bp *PRL* fragment with *XbaI* revealed only AA and GG genotypes; AG was absent with 0% of the observed heterozygosity. As shown in Figs. 1 and 2, the banding patterns of *PRL* gene yielded in PCR product were one band in AA genotype with fragment length of 678 *bp* and two bands in GG genotype with fragment length of 447 and 231 *bp*. Across the two studied buffalo herds, as shown in Table 3, the genotypic frequency of AA genotype of *PRL* gene was high (0.851) and the frequency of GG genotype was low (0.149). Also, the allelic frequency recorded for A allele was higher than that recorded for G allele (0.851 vs. 0.149). In comparing the NG herd with NK herd, the frequencies of AA and GG genotypes of *PRL* gene were similar (0.900 vs. 0.845 for AA genotype; 0.100 vs. 0.155 for GG genotype; Table 3).

**Table 3:** Molecular characterization parameters for *PRL* gene in NG and NK herds in Egyptian Buffalo

Item	NG herd	NK herd	Both herds
Observed number of animals in each PRL gene genotype			
AA	27	60	86
AG	ND	ND	ND
GG	3	11	15
Total number of genotyped animals (observed)	30	71	101
Expected number of animals in each PRL gene genotype			
AA	24.3	50.4	73.2
AG	5.4	18.6	25.6
GG	0.3	1.7	2.2
Total number of genotyped animals (expected)	30	71	101
Genotypic frequency:			
AA	0.900	0.845	0.851
AG	ND	ND	ND
GG	0.100	0.155	0.149
Gene frequency:			
A allele	0.900	0.845	0.851
G allele	0.100	0.155	0.149
Effective number of alleles (*Ne*)	1.220^b^	1.355^a^	1.339
Chi-square value for Hardy-Weinberg equilibrium (*χ2*)	30^a^	71^b^	101***
Polymorphic information content (*PIC*)	0.157	0.223	0.211
Observed heterozygosity (*H*_*O*_)	0.0	0.0	0.0
Expected heterozygosity (*H*_*E*_)	0.180	0.262	0.253

^a, b^ The estimate with the same letters in each column are not significantly different (*P* ≤ 0.01); ND = Not detected

The effective numbers of alleles (*N*_*e*_) as an index of genetic diversity revealed that the difference in *N*_*e*_ between NG and NK herds was significant (1.220 vs. 1.355, *P* < 0.01; Table 3). Chi-square values (χ^2^) for genotypes of *PRL* gene were highly significant in NG and NK herds (Table 3). The current *PIC* values were low and varied from 0.157 in NG herd to a moderate value of 0.223 in NK herd and moderate value of 0.211 in both herds (Table 3). The expected heterozygosity (*H*_*E*_) values for *PRL* gene were moderate with values of 0.180 in NG herd, 0.262 in NK and 0.253 in both herds together (Table 3).

### Polymorphic characterization of *DGAT1* gene

**Fig. 3:**
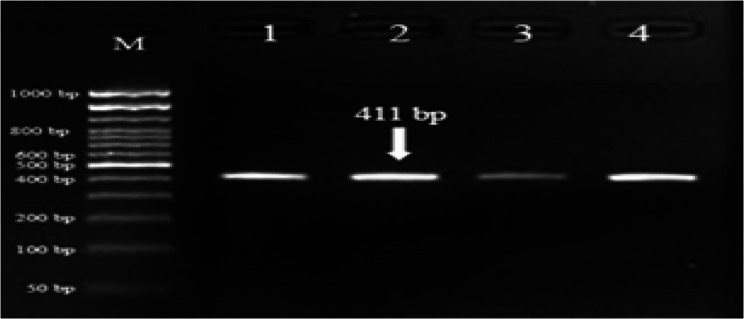
Gel electrophoresis showing the PCR product of *DGAT1* gene in Egyptian buffalo, 411 *bp* band. M is 50 *bp* ladder DNA molecular marker

**Fig. 4:**
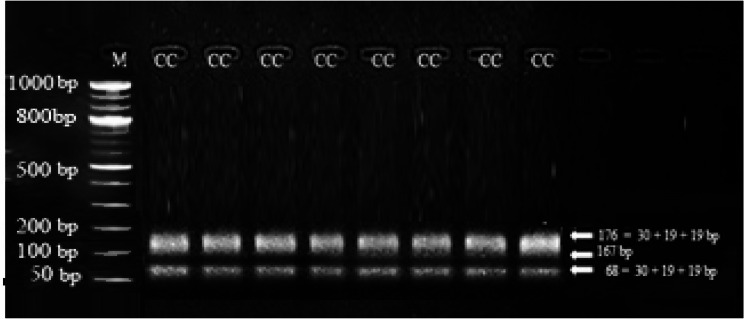
Gel electrophoresis showing the PCR-RFLP of the *SNP* identified in *DGAT1* gene in Egyptian buffalo. The genotypes are indicated at the top of each lane, 176 *bp*, 167 *bp* and 68 *bp* bands. M is 50 *bp* ladder DNA molecular marker

The genotypic frequency of genotype CC was 100% with frequency of 1.0 for allele C and 0.0 for allele T in current herds of Egyptian buffalo. The PCR amplified DNA fragment length of 411 *bp* was digested with *AluI* restriction enzyme, where CC genotype was the only one identified for *DGAT1* gene (Figs. 3 and 4). *AluI* restriction analysis of the PCR product yielded banding pattern corresponding to one genotype of CC with three bands with fragment length of 176, 167 and 68 *bp*.

### Polymorphic characterization of *FSHR* gene

**Fig. 5:**
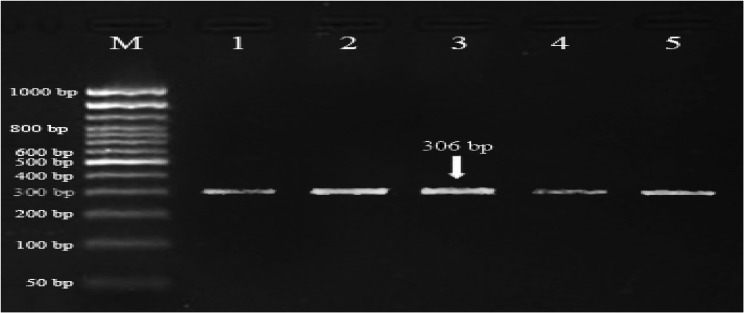
Gel electrophoresis showing the PCR product of *FSHR* gene in Egyptian buffalo, 306 *bp* band. M is 50 *bp* ladder DNA molecular marker

**Fig. 6:**
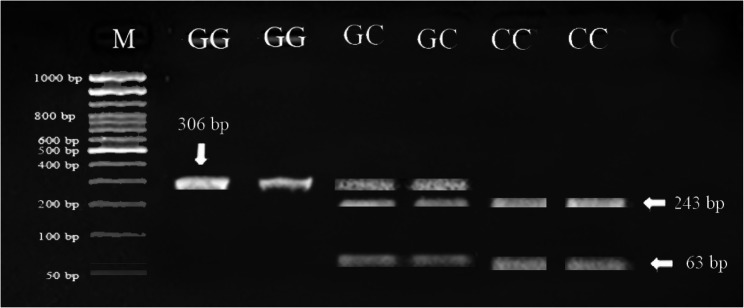
Gel electrophoresis showing the PCR-RFLP of the SNP identified of *FSHR* gene in Egyptian buffalo. The genotypes are indicated at the top of each lane, 306 *bp*, 243 *bp* and 63 *bp* bands. M is 50 *bp* ladder DNA molecular marker

Digestion of the 306 bp *FSHR* fragment with *AluI* revealed three genotypes: GG, GC, and CC. As shown in Figs. 5 and 6, the banding patterns of *FSHR* gene yielded in PCR product were one band in GG genotype (306 *bp*), two bands in CC genotype (243 and 63 *bp*) and three bands in GC genotype (306, 243 and 63 *bp*).

The genotypic frequencies for genotypes of *FSHR* gene for total buffalo cows and bulls were 0.41 for GC genotype, 0.21 for GG genotype and 0.38 for CC genotype (Table 4), i.e. allelic frequency for C allele (0.592) was higher than that for G allele (0.408). Also, the genotypic frequency for GG, GC and CC genotypes of *FSHR* gene were 0.14, 0.51 and 0.35 for buffalo cows and 0.28, 0.28 and 0.44 for buffalo bulls. The frequency of GG, GC and CC genotypes of *FSHR* gene in NG, NK and EG herds were widely differed (0.212 in NG herd, 0.113 in NK herd and 0.095 in EG herd for GG genotype; 0.515 in NG herd, 0.500 in NK herd and 0.534 in EG herd for GC genotype; 0.272 in NG herd, 0.386 in NK herd and 0.381 in EG herd for CC genotype). The frequencies for C allele were higher than those for G allele where the frequencies were 0.530, 0.636 and 0.643 for C allele vs. 0.470, 0.364 and 0.357 for G allele in NG, NK and EG herds, respectively.

**Table 4:** Molecular characterization parameters for *FSHR* gene in NG, NK and EG herds in Egyptian Buffalo

Item	NG herd	NK herd	EG herd	All herds		
				Buffalo cows	Buffalo bulls	Total (cows + bulls)
Observed number of animals in each *FSHR* gene genotype						
GG	7	5	2	14	20	34
GC	17	22	11	50	20	70
CC	9	17	8	34	31	65
Total number of genotyped animals (observed)	33	44	21	98	71	169
Expected number of animals in each *FSHR* gene genotype						
GG	7.28	5.82	2.68	15.52	12.68	28.17
GC	16.44	20.36	9.64	46.96	34.65	81.66
CC	9.28	17.82	8.68	35.52	23.68	59.17
Total number of genotyped animals (expected)	33	44	21	98	71	169
Genotypic frequency:						
GG	0.212	0.113	0.095	0.14	0.28	0.21
GC	0.515	0.500	0.534	0.51	0.28	0.41
CC	0.272	0.386	0.381	0.35	0.44	0.38
Gene frequency:						
G allele	0.470	0.364	0.357	0.40	0.42	0.408
C allele	0.530	0.636	0.643	0.60	0.58	0.592
Effective number of alleles (*Ne*)	1.993^ba^	1.862^c^	1.849^c^	1.953^b^	1.920^a^	1.936^a^
Chi-square value for *HWE* (*χ*^*2*^)	0.038^NS^	0.284^NS^	0.416^NS^	0.411^NS^	12.69^***^	3.444 ^NS^
Polymorphic information content (*PIC*)	0.531	0.653	0.662	0.609	0.586	0.600
Observed heterozygosity (*H*_*O*_)	0.515	0.500	0.524	0.510	0.282	0.414
Expected heterozygosity (*H*_*E*_)	0.498	0.463	0.459	0.479	0.488	0.483

^a, b, c^ The estimate with the same letters in each column are not significantly different (*P* ≤ 0.05); NS = Non-significant (*P* > 0.05)

The difference in the effective number of alleles (*N*_*e*_) among NG, NK and EG herds for *FSHR* gene were significant (*P* < 0.01) where *N*_*e*_ was 1.993, 1.862 and 1.849 in NG, NK and EG herds, respectively (Table 4). Also, the *N*_*e*_ were 1.953 and 1.920 in buffalo cows and bulls, respectively. The difference among genotypes of Chi-square for *FSHR* gene were not significant in NG, NK and EG herds (Table 4), indicating that all herds were in *HWE* for *FSHR* gene.

The current *PIC* values were moderate and varied from 0.531 in NG herd to 0.653 and 0.662 in NK and EG herds, respectively (Table 4). The values of expected heterozygosity (*H*_*E*_) for *FSHR* gene were high, being 0.498 in NG herd, 0.463 in NK herd, 0.459 in EG herd, 0.479 in buffalo cows and 0.488 in buffalo bulls, while the observed heterozygosity (*H*_*O*_) was 0.515 in NG herd, 0.500 in NK herd, 0.524 in EG herd, 0.510 in buffalo cows and 0.282 in buffalo bulls (Table 4).

### Polymorphic characterization of *GH* gene

**Fig. 7:**
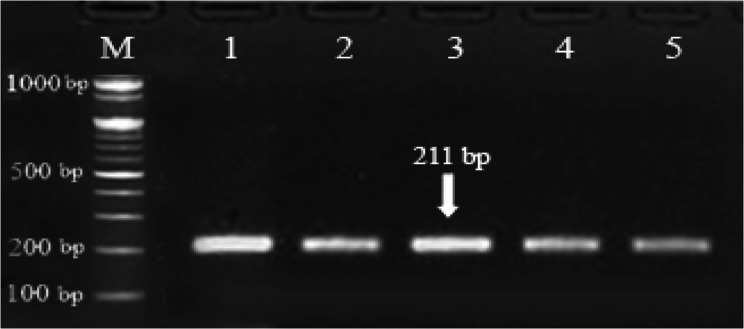
Gel electrophoresis showing the PCR product of *GH* gene in Egyptian buffalo, 211 *bp* band. M is 100 bp ladder DNA molecular marker

**Fig. 8:**
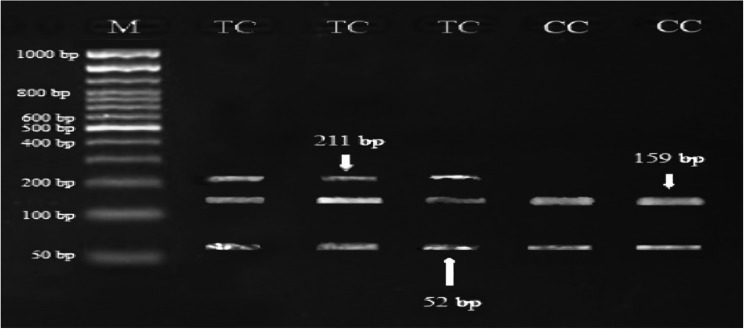
Gel electrophoresis showing the PCR-RFLP of the SNP identified of *GH* gene in Egyptian buffalo. The genotypes are indicated at the top of each lane, 211 *bp*, 159 *bp* and 52 *bp* bands. M is 50 *bp* ladder DNA molecular marker

Digestion of 211 bp of *GH* fragment with revealed two genotypes: CC and TC. The PCR product using *AluI* restriction enzyme yielded banding pattern corresponding to two bands of 159 and 52 *bp* for CC genotype and three bands of 211, 159 and 52 *bp* for TC genotype (Figs. 7 and 8). The frequencies of CC genotype for *GH* gene were 0.608 in NG herd, 0.505 in NK herd and 0.500 in EG herd, while the frequencies of TC genotype were 0.392 in NG herd, 0.495 in NK herd and 0.500 in EG herd (Table 5). Across all the herds, the frequencies of CC genotype were 0.68 for females, 0.30 for males and 0.52 for both sexes, while the frequencies of TC genotype were 0.32 for females, 0.70 for males and 0.48 for both sexes. The frequencies recorded for C allele (0.804 in NG herd, 0.753 in NK herd and 0.750 in EG herd) were higher than those recorded for T allele (0.196 in NG herd, 0.247 in NK herd and 0.250 in EG herd). The effective numbers of alleles (*N*_*e*_) and chi-square values characterizing *GH* gene in each herd are presented in Table 5. The difference in allelic numbers among the three herds were significant (*P* < 0.01). Across all herds, the highest *Ne* was obtained for males (1.839), while the lowest allelic numbers was obtained for females (1.368). The Chi-square values for genotypes of *GH* gene were not significant in females and highly significant in males (Table 5), indicating that this population was in *HWE* for *GH* gene. Across the herds and sexes, the values of heterozygosity for *GH* gene were moderate to high and ranged from 0.320 to 0.704 for *Ho* and 0.269 to 0.456 for *H*_*E*_ (Table 5).

**Table 5:** Molecular characterization parameters for *GH* gene in NG, NK and EG herds in Egyptian Buffalo

Item	NG herd	NK herd	EG herd	All herds		
				Females	Males	Total (females + males)
Observed number of animals in each *GH* gene genotype						
TT	ND	ND	ND	ND	ND	ND
TC	20	48	13	33	50	83
CC	31	49	13	70	21	91
Total number of genotyped animals (observed)	51	97	26	103	71	174
Expected number of animals in each *GH* gene genotype						
TT	1.96	0.594	1.63	2.64	8.80	9.90
TC	16.08	36.12	9.75	27.71	32.39	63.20
CC	32.96	45.94	14.63	72.64	29.80	100.9
Total number of genotyped animals (expected)	51	97	26	103	71	174
Genotypic frequency:						
TT	ND	ND	ND	ND	ND	ND
TC	0.392	0.495	0.500	0.32	0.70	0.48
CC	0.608	0.505	0.500	0.68	0.30	0.52
Gene frequency:						
T allele	0.196	0.247	0.250	0.160	0.352	0.352
C allele	0.804	0.753	0.750	0.840	0.648	0.648
Effective number of alleles (*Ne*)	1.460^c^	1.593^b^	1.600^a^	1.368^b^	1.839^a^	1.570
Chi-square value for *HWE* (*χ*^*2*^)	3.034^ns^	10.485^**^	2.889^ns^	3.748^ns^	20.97^***^	17.069^***^
Polymorphic information content (*PIC*)	0.862	0.801	0.797	0.902	0.668	0.810
Observed heterozygosity (*H*_*O*_)	0.392	0.495	0.500	0.320	0.704	0.704
Expected heterozygosity (*H*_*E*_)	0.315	0.372	0.375	0.269	0.456	0.456

^a, b^ The estimate with the same letters in each row are not significantly different (*P* ≤ 0.05); NS = Non-significant (*P* > 0.05), *** = *p* < 0.001; ND = Not detected

### Molecular associations between genotypes of *PRL* gene and lactation or reproduction traits

Two genotypes of AA and GG for *PRL* gene were detected (Table 6). For lactation traits, the GLSM for SNP genotypes of *PRL* gene showed that there were molecular associations of AA and GG genotypes with test-day milk traits (Table 6). The differences in GLSM for lactation traits between AA and GG genotypes of *PRL* gene in NG and NK herds were significantly in favour of AA genotype (*P* < 0.01, Table 6). In both NG and NK herds, high GLSM were recorded for AA genotype to be 6.0 kg for TDMY, 390 g for TDFY, 290 g for TDPY and 2.47 for TDSCS compared with 5.3 kg, 340 g, 220 g and 2.50 for GG genotype, respectively. In NG herd, GLSM for lactation traits were significantly in favour of AA genotype of *PRL* gene relative to GG genotype in terms of 5.9 vs. 5.5 kg for TDMY, 360 vs. 310 g for TDFY, 260 vs. 220 g for TDPY and 2.38 vs. 2.52 for TDSCS, while the respective GLSM in NK herd were 5.97 vs. 5.43 kg, 390 vs. 350 g, 290 vs. 230 g and 2.41 vs. 2.49.

**Table 6:** Molecular associations between genotypes of *PRL* gene (AA and GG genotypes) and test-day lactation traits or reproduction performance expressed as generalized least square means and their standard errors (GLSM ± SE)

Herd and lactation trait	AA Genotype	GG Genotype	Herd and reproduction trait	AA Genotype	GG Genotype
	GLSM ± SE	GLSM ± SE		GLSM ± SE	GLSM ± SE
**NG herd**:	**(** ***N*** ** = 282)**	**(** ***N*** ** = 42)**	**NG herd**:	**(** ***N*** ** = 125)**	**(** ***N*** ** = 18)**
TDMY (kg)	5.9 ± 0.09^a^	5.5 ± 0.25^b^	AFC (*mo*)	43.0 ± 0.4^a^	41.0 ± 1.2^b^
TDFY (kg)	0.36 ± 0.01^a^	0.31 ± 0.02^b^	DO (*d*)	174 ± 9.4^a^	142 ± 24.9^b^
TDPY (kg)	0.26 ± 0.04^a^	0.22 ± 0.01^b^	CI (*d*)	476 ± 9.4^a^	435 ± 24.8^b^
TDSCS	2.38 ± 0.03^b^	2.52 ± 0.01^a^			
**NK herd**:	**(** ***N*** ** = 590)**	**(** ***N*** ** = 115)**	**NK herd**:	**(** ***N*** ** = 162)**	**(** ***N*** ** = 48)**
TDMY (kg)	5.97 ± 0.09^a^	5.43 ± 0.20^b^	AFC (*mo*)	35.1 ± 0.3ª	33.8±0.6 ^b^
TDFY (kg)	0.39 ± 0.01^a^	0.35 ± 0.01^b^	DO (*d*)	158 ± 8.1ª	143 ± 14.9^b^
TDPY (kg)	0.29 ± 0.04^a^	0.23 ± 0.04^b^	CI (*d*)	459 ± 7.9^a^	449 ± 14.6^b^
TDSCS	2.41 ± 0.03^b^	2.49 ± 0.08^a^			
**Both herds**:	**(** ***N*** ** = 872)**	**(** ***N*** ** = 157)**	**EG herd**:	**(** ***N*** ** = 82)**	**(** ***N*** ** = 18)**
TDMY (kg)	6.0 ± 0.06^a^	5.3 ± 0.15^b^	AFC (*mo*)	37.4 ± 0.4ª	34.8 ± 0.9^b^
TDFY (kg)	0.39 ± 0.01^a^	0.34 ± 0.01^b^	DO (*d*)	185 ± 23.0ª	170 ± 11.6^b^
TDPY (kg)	0.29 ± 0.01^a^	0.22 ± 0.01^b^	CI (*d*)	481 ± 23.1^a^	469 ± 11.6^b^
TDSCS	2.47 ± 0.01^b^	2.50 ± 0.02^a^			
			**All herds**:	**(** ***N*** ** = 369)**	**(** ***N*** ** = 84)**
			AFC (*mo*)	37.4 ± 0.2^a^	36.5 ± 0.6^b^
			DO (*d*)	166 ± 5.3^a^	154 ± 11.2^b^
			CI (*d*)	467 ± 5.3^a^	455 ± 11.1^b^

N = Number of test-day lactation records or number of reproduction records

^a, b^ GLSM within each classification, not followed by the same letter in the row differed significantly (*P* < 0.01)

For most reproduction traits, GLSM for AA and GG genotypes of *PRL* gene showed that there were significant molecular associations between AA and GG genotypes with AFC, DO and CI (Table 6). Also, the differences in GLSM for AFC, DO and CI between AA and GG genotypes of *PRL* gene were significantly in favour of GG genotype relative to AA genotype in NG, NK and EG herds (*P* < 0.01), i.e. GLSM for GG genotype ranging from 33.8 to 41 *mo* for AFC, 142 to 170 *d* for DO and 435 to 469 *d* for CI. In NG herd, GLSM for GG genotype of *PRL* gene were significantly favourable for reproduction traits compared to AA genotype in terms of 41.0 vs. 43.0 *mo* for AFC, 142 vs. 174 *d* for DO and 345 vs. 476 *d* for CI, while the corresponding GLSM in NK were 33.8 vs. 35.1 *mo*, 143 vs. 158 *d* and 449 vs. 459 *d*.

### Molecular associations between genotypes of *FSHR* gene and lactation or reproduction traits

The differences in GLSM for lactation traits among GG, GC and CC genotypes of *FSHR* gene in the three herds studied were significantly in favour of CC genotype (*P* < 0.01, Table 7). GLSM for lactation traits in NG herd were significantly in favour of CC genotype of *FSHR* gene relative to GC and GG genotypes in terms of 6.8 kg vs. 5.7 and 6.0 kg for TDMY, 480 g vs. 380 and 410 g for TDFY, 280 g vs. 220 and 250 g for TDPY and 2.41 vs. 2.43 and 2.49 for TDSCS, while the corresponding GLSM in NK herd were 6.8 kg vs. 5.4 and 5.5 kg, 390 g vs. 340 and 320 g, 290 g vs. 250 and 230 g and 2.41 vs. 2.45 and 2.49.

**Table 7:** Molecular associations between genotypes of *FSHR* gene (GG, GC and CC genotypes) and lactation traits expressed as generalized least square means and their standard errors (GLSM ± SE)

Herd and lactation trait	GG Genotype	GC Genotype	CC Genotype
	GLSM ± SE	GLSM ± SE	GLSM ± SE
**NG herd:**	**(** ***N*** ** = 107)**	**(** ***N*** ** = 212)**	**(** ***N*** ** = 116)**
TDMY (kg)	6.0 ± 0.186^b^	5.7 ± 0.132^c^	6.8 ± 0.179^a^
TDFY (kg)	0.41 ± 0.016^c^	0.38 ± 0.012^b^	0.48 ± 0.015^a^
TDPY (kg)	0.25 ± 0.008^b^	0.22 ± 0.005^b^	0.28 ± 0.007ª
TDSCS	2.49 ± 0.026^a^	2.43 ± 0.019^b^	2.41 ± 0.025^b^
**NK herd**:	**(** ***N*** ** = 87)**	**(** ***N*** ** = 392)**	**(** ***N*** ** = 288)**
TDMY (kg)	5.5 ± 0.232^b^	5.4 ± 0.109^a^	6.8 ± 0.127^a^
TDFY (kg)	0.32 ± 0.016^b^	0.34 ± 0.007^b^	0.39 ± 0.009ª
TDPY (kg)	0.23 ± 0.010^c^	0.25 ± 0.005^b^	0.29 ± 0.006ª
TDSCS	2.49 ± 0.025^a^	2.45 ± 0.012^b^	2.41 ± 0.014^c^
**Both herds**:	**(** ***N*** ** = 194)**	**(** ***N*** ** = 604)**	**(** ***N*** ** = 404)**
TDMY (kg)	5.6 ± 0.139^b^	5.7 ± 0.078^b^	6.8 ± 0.096^a^
TDFY (kg)	0.36 ± 0.007^a^	0.35 ± 0.006^b^	0.38 ± 0.010^a^
TDPY (kg)	0.22 ± 0.006^c^	0.23 ± 0.003^b^	0.29 ± 0.004^a^
TDSCS	2.48 ± 0.017^a^	2.45 ± 0.010^b^	2.41 ± 0.012^c^

*N* = Number of test-day lactation records

^a, b^ GLSM within each classification, not followed by the same letter in the row differed significantly (*P* < 0.01)

The differences in GLSM for reproduction traits among GG, GC and CC genotypes of *FSHR* gene were significantly in favour of CC genotype (*P* < 0.01, Table 8). The GLSM recorded for CC genotype of *FSHR* gene were significantly favourable lower than GLSM for GC and GG genotypes in terms of 37.9 *mo vs.* 39.7 and 42.5 *mo* for AFC, 83 *d vs.* 91 and 102 *d* for DO and 387 *d vs.* 397 and 419 *d* for CI in NG herds, comparable with 32.0 *mo vs.* 34.3 and 35.6 *mo* for AFC, 83 *d vs.* 91 and 102 *d* for DO and 384 *d vs.* 408 and 398 *d* for CI in NK herd (Table 8). Also, favourable trends were observed in EG herd where GLSM were 35.0 *mo vs.* 37.3 and 36.5 *mo* for AFC, 103 *d vs.* 109 and 118 *d* for DO and 366 *d vs.* 396 and 410 *d* for CI.

**Table 8:** Molecular associations between genotypes of *FSHR* gene (GG and GC genotypes) and reproduction traits expressed as generalized least square means and their standard errors (GLSM ± SE)

Herd and reproduction trait	GG Genotype	GC Genotype	CC Genotype
	GLSM ± SE	GLSM ± SE	GLSM ± SE
**NG herd:**	**(** ***N*** ** = 37)**	**(** ***N*** ** = 76)**	**(** ***N*** ** = 38)**
AFC (*mo*)	42.5 ± 0.78^a^	39.7 ± 0.51^b^	37.9 ± 0.73^c^
DO (*d*)	102 ± 13.9^a^	91 ± 9.7^b^	83 ± 13.8^c^
CI (*d*)	419 ± 15.3^a^	397 ± 10.7^b^	387 ± 15.16^c^
**NK herd**:	**(** ***N*** ** = 28)**	**(** ***N*** ** = 114)**	**(** ***N*** ** = 80)**
AFC (*mo*)	35.6 ± 0.85^a^	34.3 ± 0.42^b^	32.0 ± 0.50^c^
DO (*d*)	102 ± 14.7^a^	91 ± 7.3^b^	83 ± 8.7^c^
CI (*d*)	398 ± 15.9^b^	408 ± 7.9 ^a^	384 ± 9.4^c^
**EG herd**:	**(** ***N*** ** = 9)**	**(** ***N*** ** = 39)**	**(** ***N*** ** = 36)**
AFC (*mo*)	36.5 ± 1.36^b^	37.3 ± 0.65^a^	35.0 ± 0.68^c^
DO (*d*)	118 ± 26.4^a^	109 ± 12.7^b^	103 ± 13.2^c^
CI (*d*)	410 ± 29.1^a^	396 ± 14.0^b^	366 ± 14.5^c^
**All herds**:	**(** ***N*** ** = 74)**	**(** ***N*** ** = 240)**	**(** ***N*** ** = 154)**
AFC (*mo*)	38.4 ± 0.61^a^	37.6 ± 0.33^b^	34.5 ± 0.42^c^
DO (*d*)	115 ± 9.1^a^	108 ± 5.0^b^	100 ± 6.3^c^
CI (*d*)	411 ± 9.8^a^	402 ± 5.4^b^	391 ± 6.8^c^

*N* = Number of reproduction records

^a, b^ GLSM within each classification, not followed by the same letter in the row differed significantly (*P* < 0.01)

### Molecular associations between genotypes of *GH* gene and lactation or reproduction traits

Two genotypes of TC and CC for *GH* gene in each NG and NK separate herds were significantly in favour of TC genotype for lactation traits (*P* < 0.01, Table 9). The GLSM for TC genotype of *GH* gene in NG herd were significantly higher in lactation traits than that of CC genotype in terms of 6.3 vs. 5.8 kg for TDMY, 480 vs. 380 g for TDFY and 290 vs. 230 g for TDPY (Table 9). Also, favourable respective GLSM of 6.3 vs. 5.6 kg, 390 vs. 350 g and 290 vs. 230 g were confirmed in NK herd. Moreover, GLSM of TDSCS were in favour of TC genotype relative to CC genotype (2.41 and 2.45 in NG and NK herds).

**Table 9:** Molecular associations between genotypes of *GH* gene (TC and CC genotypes) and lactation and reproduction traits expressed as generalized least square means and their standard errors (GLSM ± SE)

Herd and lactation trait	TC Genotype	CC Genotype	Herd and reproduction trait	TC Genotype	CC Genotype
	GLSM ± SE	GLSM ± SE		GLSM ± SE	GLSM ± SE
**NG herd:**	**(** ***N*** ** = 98)**	**(** ***N*** ** = 355)**	**NG herd:**	**(** ***N*** ** = 106)**	**(** ***N*** ** = 37)**
TDMY (kg)	6.3 ± 0.19ª	5.8 ± 0.10^b^	AFC (*mo*)	37.8 ± 0.79^b^	41.4 ± 0.47^a^
TDFY (kg)	0.48 ± 0.02ª	0.38 ± 0.08^b^	DO (*d*)	93 ± 14.1^b^	115 ± 8.3ª
TDPY (kg)	0.29 ± 0.01ª	0.23 ± 0.04^b^	CI (*d*)	383 ± 15.6^b^	407 ± 9.2ª
TDSCS	2.41 ± 0.03^b^	2.45 ± 0.01^a^			
**NK herd**:	**(** ***N*** ** = 297)**	**(** ***N*** ** = 481)**	**NK herd**:	**(** ***N*** ** = 154)**	**(** ***N*** ** = 70)**
TDMY (kg)	6.3 ± 0.12^a^	5.6 ± 0.09^b^	AFC (*mo*)	33.7 ± 0.54^b^	35.2 ± 0.36ª
TDFY (kg)	0.39 ± 0.01ª	0.35 ± 0.01^b^	DO (*d*)	94 ± 9.4^b^	100 ± 6.3^a^
TDPY (kg)	0.29 ± 0.01ª	0.23 ± 0.04^b^	CI (*d*)	379 ± 10.2^b^	393 ± 6.9^a^
TDSCS	2.41 ± 0.01^b^	2.45 ± 0.01^b^			
**Both herds**:	**(** ***N*** ** = 395)**	**(** ***N*** ** = 836)**	**EG herd**:	**(** ***N*** ** = 59)**	**(** ***N*** ** = 34)**
TDMY (kg)	6.1 ± 0.10ª	5.6 ± 0.07^b^	AFC (*mo*)	35.4 ± 0.65^b^	37.5 ± 0.49ª
TDFY (kg)	0.39 ± 0.01ª	0.35 ± 0.01^b^	DO (*d*)	105 ± 13.4^b^	121 ± 10.2^a^
TDPY (kg)	0.29 ± 0.04ª	0.22 ± 0.01^b^	CI (*d*)	395 ± 15.0^b^	406 ± 11.4ª
TDSCS	2.41 ± 0.01^b^	2.45 ± 0.01^b^			
			**All herds**:	**(** ***N*** ** = 326)**	**(** ***N*** ** = 145)**
			AFC (*mo*)	34.4 ± 0.44^b^	37.6 ± 0.29^a^
			DO (*d*)	95 ± 6.7^b^	107 ± 4.5ª
			CI (*d*)	377 ± 7.4^b^	399 ± 4.9ª

*N* = Number of test-day lactation records or number of reproduction records

^a, b^ GLSM within each classification, not followed by the same letter in the row differed significantly (*P* < 0.01)

For the molecular association between the genotypes of *GH* gene with reproduction traits in NG, NK and EG herds, the differences between TC and CC genotypes were significantly in favour of TC genotype (Table 9). The GLSM recorded for TC genotype in NG, NK and EG herds were significantly the lowest favourable genotypes for AFC (37.8, 33.7 and 35.4 *mo*), DO (93, 94 and 105 *d*) and CI (383, 379 and 395 *d*), comparable with the corresponding GLSM of 41.4, 35.2 and 37.5 *mo* for AFC, 115, 100 and 121 *d* for DO and 407, 393 and 406 *d* for CI.

## Discussion

Regarding the polymorphic characterization of *PRL* gene, Hasanain et al. [18] identified only one band of fragment length of 678 *bp* for AA genotype in Egyptian buffalo. Mavi et al. [14] in Murrah buffalo found one genotype of AA for *PRL* gene with fragment length of 294 *bp*. Konca and Akyüz [15] reported that the undigested fragment of 156 *bp* for *PRL* gene in Anatolian water buffalo refer to AA genotype, while the fragments of 156, 82 and 74 *bp* indicated for heterozygous genotype. The absence of AG accompanied with 0% of the observed heterozygosity may be attributed to many factors such as inbreeding depression, genetic drift, strong artificial selection and/or historical bottlenecks in the studied buffalo populations. Also, in Anatolian water buffalo, Özşensoy [16] reported that the undigested fragment of 156 *bp* for *PRL* gene refer to AA genotype, while the fragments of 156, 82 and 74 *bp* refer to heterozygous genotype. Ladani et al. [46] stated that the frequencies of A allele for *PRL* gene in Jaffarabadi, Mehsani and Surti buffaloes were 0.43, 0.50 and 0.48, respectively. Ishaq et al. [13] examined *PRL* gene polymorphisms in Sahiwal and Achai buffalo using PCR-RFLP technique and reported that three genotypes of AA, AG and GG were detected with frequencies of 0.72, 0.18 and 0.10 in Sahiwal buffalo and 0.44, 0.34 and 0.22 in Achai buffalo, respectively. El-Magd et al. [7] in Egyptian buffalo found two genotypes for *PRL* gene and reported that the genotypic frequencies were 0.37 for CC genotype and 0.63 for CT genotype and accordingly the allele frequency was 0.315 for C allele and 0.685 for T allele. They added that *Ne* was 1.759 and this moderate value reflected moderate genetic diversity, polymorphism, and ability to preserve allelic stability after selection or mutation. In accordance, Konca and Akyüz [15] showed that the value of Chi-square for genotypes of *PRL* gene in Anatolian water buffalo was high (50.63), indicating that this population was not in *HWE*. In the present study, both populations were not in *HWE* for *PRL* gene, *i.e.* degree of variation between the numbers of the expected and observed genotypes was high. This high deviation in *HWE* suggests the change in distribution of alleles from one generation to the next generations. In addition to the impact of limited number of bulls used as a breeding practice in these populations, genetic drift in these small populations and/or strong artificial selection. Depending on the number of detectable alleles and the distribution of their frequency, the value of *PIC* gives an estimate of the marker’s discriminating power and, thus, describes the marker’s usefulness for identifying the polymorphism within the buffalo population under study [7].

In the current study, *DGAT1* SNP was monomorphic (100% CC) and therefore not informative for association with the studied traits in these buffalo populations, this result impairs its use in marker-assisted selection programs within this genetic background. However, *DGAT1* gene is known to control the rate of triglyceride synthesis via adipocytes and consequently influence the fatty acids contents in milk [47–49] and it was verified to have associations with lactation and/or reproduction traits in Chinese buffalo [49], in Anatolian buffalo [19], in Murrah buffalo [21] and in Iraqi buffalo [22]. Concerning the molecular characterization of *DGAT1* gene, Yuan et al. [47] in Chinese buffalo reported that the range in band size of *DGAT1* gene was from 160 *bp* to 300 *bp*. In agreement with the current findings, Ozdil and Ilhan [19] in Anatolian buffalo reported that the undigested fragment with 411 *bp* for *DGAT1* gene refer to GG genotype, while the digested fragments of 176, 167 and 68 *bp* refer to CC genotype and the fragments of 411, 167, 137 and 107 *bp* were indicated for heterozygous GC genotype. Freitas et al. [3] showed that the PCR fragment size was 231 *bp* for *DGAT1* gene in Murrah buffalo.

For the molecular characterization of *FSHR* gene, Othman and Abd-El Samad [23] in PCR amplified fragments (306 *bp*) and using *AluI* restriction enzyme in Egyptian buffalo identified three genotypes of *FSHR* gene (CC, CG and GG), indicating that two bands of 243 and 63 *bp* for CC genotype, three bands of 193, 63 and 50 *bp* for GG genotype and four bands of 243, 193, 63 and 50 *bp* for CG genotype. By using the same restriction enzyme for digestion of 306 *bp* PCR product, Sosa et al. [50] differentiated between three genotypes for *FSHR* gene in Egyptian buffalo (CC, TT and CT) and reported that two bands with fragments length of 243 and 63 *bp* for CC genotype, three bands of 193, 63 and 50 *bp* for GG genotype and four bands of 243, 193, 63 and 50 *bp* for CG genotype were identified. Shafik et al. [34] showed that one non-synonymous *SNP* (A93G) was detected in Egyptian buffalo in exon 10 of *FSHR* gene with fragment length of 230 *bp*. Recently, Dhaware et al. [51] reported that the third fragment of exon 10 of *FSHR* gene was amplified by using forward primer *FSHR3f* and reverse primer *FSHR3r* revealing a PCR product of fragment length 910 bp in Indian Marathwadi buffalo.

In Egyptian buffalo, Shafik et al. [34] found that the frequencies for A and G alleles of *FSHR* gene were 0.014 and 0.985 along with frequencies of 0.00, 0.028 and 0.972 for AA, AG and GG genotypes, respectively. Fouda et al. [38] reported that the frequencies for C and G alleles were 0.54 and 0.46 with frequencies of 0.34, 0.40 and 0.26 for CC, CG and GG genotypes, respectively. The authors mentioned that Chi-square values of genotypes (GG and CG) for *FSHR* gene in Egyptian buffalo were moderate (X^2^ = 3.948 vs. 7.852), indicating that this population was not in *HWE*. In Indonesian Holstein dairy cattle, Setyorini and kurnianto [52] reported that the value of Chi-square for genotypes of *FSHR* gene was high (3.2), *i.e.* *FSHR* gene not was in *HWE* in this population. In this respect, Putra et al. [53] stated that the PIC values for bovine *FSHR* gene in Indonesian Pasundan cattle were moderate and ranged from 0.30 to 0.50. Moreover, in Zebu × British composite crossbred cattle and indigenous Turkish breed, the PIC values were also moderate, being 0.37 and 0.34, respectively [54, 55]. Setyorini and kurnianto [52] in Indonesian Holstein dairy cattle found that the value of *Ho* for *FSHR* gene was 0.490, while the value of *He* was 0.416.

Concerning the molecular characterization of *GH* gene, Konca and Akyüz [15] reported that fragment of 211 *bp* for *GH* gene was observed in Anatolian water buffalo. Konca and Akyüz [15] reported that the allele frequency in Anatolian buffalo was 0.87 for L allele and 0.13 for V allele, while the frequencies were 0.755, 0.228 and 0.017 for LL, LV and VV genotypes, respectively. Eriani et al. [29] stated that the frequency of *GH* gene in Indonesian buffalo was 0.533 for A allele and 0.467 for B allele, with genotypic frequencies of 0.133, 0.866 and 0.066 for AA, AB and BB genotypes, respectively. Anggraeni et al. [56] stated that the genotypic frequency of genotypes of *GH* gene in Indonesian Swamp buffalo was 100% for TT genotype and 0% for both TC and CC genotypes, with allelic frequency of 1.0 for T allele and 0.0 for C allele. In this regard, Trakovická et al. [57] found that *N*_*e*_ for *GH* gene in Slovak Simmental cattle was 1.73. The Chi-square values for genotypes of *GH* gene Similarly, Konca and Akyüz [15] reported that the value of Chi-square for genotypes of *GH* gene was low (0.02), indicating that *GH* gene in Anatolian water buffalo was in *HWE*. Nafiu et al. [28] in Swamp buffalo, stated that Chi-square value for genotypes of *GH* gene was also low (0.89), suggesting that the population was in *HWE*. Eriani et al. [29] in Indonesian buffalo found that the value of *Ho* was high (0.80) and the value of *He* was moderate (0.49). Also, Nafiu et al. [28] in Swamp buffalo found that *Ho* value was moderate (0.375), while the value of *He* was high (0.492). In the present study, a significant deviation from Hardy-Weinberg equilibrium was observed in males but not females. This may be due to that the number of genotyped males is smaller than the number of genotyped females, sex-biased selection, differential mortality and/or sampling bias in breeding bulls.

PRL hormone has several biological functions related to reproduction, osmoregulation, integument growth, and synergism with steroids because *PRL* gene is expressed in the pituitary gland as well as at various other locations, such as the mammary gland, the central nervous system, and the immune system [58, 59]. Lazebnaya et al. [60] reported that the interaction of bovine *PRL* gene and its receptor (*PRLR*) following its expression starts a signalling cascade that triggers the transcription of other several genes, including those pertaining to milk proteins such as caseins and lactalbumin. *PRL* gene is known to have various biological functions such as water and electrolyte balance, growth and development, immune and reproduction function [32, 61]. Also, *PRL* gene plays a vital role in mammalian reproduction, glandular development, milk secretion, and expression of milk protein. Several reports highlighted that *PRL* gene is associated with milk production and composition in Pakistan buffalo [4], Chinese buffalo [30], Turkish buffalo [15, 16], Indian buffalo [14] and Egyptian buffalo [7]. In Murrah buffalo, Singh et al. [33] found that *PRL* gene is an important candidate gene known to be associated with milk production traits as well as somatic cell score.

*FSHR* gene has 9 introns and 10 exons [62]. Follicle stimulating hormone drives the growth, differentiation, maturity, and ovulation of ovarian follicles by attaching this gene to its receptor (*FSHR*) on the surface of the ovary [63]. For polymorphism of genes related to reproduction traits in buffalo, *FSHR* gene is essential for follicle growth, development, differentiation, triggering the maturation and ovulation of ovarian follicles. Dhaware et al. [51] stated that *FSHR* plays a critical role in the development of anatomical, functional, and behavioural qualities required for buffalo reproduction. Shafik et al. [34] reported significant association between *FSHR* gene and total milk yield and 305-day milk yield in Egyptian buffalo. The GLSM for reproduction traits were in accordance with those means previously reported by several Egyptian investigators for Egyptian buffalo studies have shown that *FSHR* gene is considered as an important candidate gene for reproduction and fertility traits in Egyptian buffalo [23, 24, 31, 34].

The *GH* gene is thought to be a positional and functional candidate gene for ruminant qualities that have economic significance, like growth, carcass, and milk features [64]. This gene produces the anabolic hormone *GH* protein, which is produced by the anterior pituitary’s somatotrophic cells [65]. *GH*, as a key component of the somatotrophic axis, is essential for growth, reproduction, and breastfeeding primarily via promoting cell division, the synthesis of proteins and lipids, and metabolism [66]. *GHR*, the receptor for growth hormone, is expressed in a number of organs, most notably the muscles, adipose tissues, and liver [67]. Growth hormone acts by binding to this receptor. Furthermore, *GH* gene can be used as a candidate gene in the genetic improvement programs for growth traits in buffalo, since this gene is known to have various biological functions such as water and electrolyte balance and milk production [8, 68].

In practise, as stated before, GLSM for AA genotype of *PRL* gene was superior relative to GG genotype, with values of 6.0 vs. 5.3 kg for TDMY. This represents a 0.7 kg per day increase in total daily milk yield per buffalo cow, which translates to approximately 161 kg more (considering a lactation period length of 230 days). Assuming an average farm-gate milk price of $0.62 per kg, this gain corresponds to an additional $69.78 per buffalo cow per lactation. For a herd of 100 buffaloes, selection for AA genotype could yield extra revenue of $6,987 per lactation cycle. These findings highlight the potential economic benefits of incorporating PRL genotyping in marker-assisted selection (MAS) programs for improving both productivity and profitability in buffalo herds.

## Conclusions

Based on the significant molecular associations detected in the present study, AA and GG genotypes of *PRL* gene, CC genotype of *FSHR* gene and TC genotype of *GH* gene may be advantageous for marker-assisted selection programs aiming to improve lactation traits (milk, fat, protein and somatic cell score) and reproduction performance (age at first calving, days open and calving interval) in Egyptian buffalo. Although the present results had shown that there was an association between *PRL*, *FSHR* and *GH* genes with lactation and reproduction traits, it is unknown whether the current DNA polymorphisms of these genes are the causative variant or just molecular markers linked with the real mutation. Therefore, further studies such as linkage analysis and functional genomics are necessary to validate these SNP-trait associations in larger and more genetically diverse buffalo populations and to elucidate the regulatory mechanisms of these polymorphisms, which would strengthen their candidacy for marker-assisted selection.

## Supplementary Information

Supplementary Material 1.

## Data Availability

No datasets were generated or analysed during the current study. All results of the data used were presented through the manuscript. The raw data is available on reasonable requests from the corresponding author.
